# Telerehabilitation improves cardiorespiratory and muscular fitness and body composition in older people with post‐COVID‐19 syndrome

**DOI:** 10.1002/jcsm.13530

**Published:** 2024-06-27

**Authors:** Eulogio Pleguezuelos, Amin Del Carmen, Eva Moreno, Mateu Serra‐Prat, Noemí Serra‐Payá, Manuel Vicente Garnacho‐Castaño

**Affiliations:** ^1^ Department of Physical Medicine and Rehabilitation Hospital of Mataro Barcelona Spain; ^2^ Department of Experimental Science and Healthcare. Faculty of Health Sciences Universitat Pompeu Fabra Barcelona Spain; ^3^ Department of Physical Medicine and Rehabilitation Hospitalet General Hospital, L'Hospitalet de Llobregat Barcelona Spain; ^4^ Research Unit Consorci Sanitari del Maresme Barcelona Spain; ^5^ DAFNiS Research Group (Pain, Physical Activity, Nutrition and Health) Campus Docent Sant Joan de Déu, Universitat de Barcelona Barcelona Spain; ^6^ Facultad de Ciencias de la Salud Universidad Internacional de Valencia (VIU) Valencia Spain

**Keywords:** Exercise capacity, Muscle strength, Peak oxygen uptake, Randomized‐controlled trial, Skeletal muscle mass, Ventilatory efficiency

## Abstract

**Background:**

The effects of post‐coronavirus disease 2019 (COVID‐19) syndrome on the cardiorespiratory and muscular fitness in older people are of utmost relevance. This study aimed to evaluate the effects of a 12‐week telerehabilitation programme on cardiorespiratory and muscular fitness and body composition in older patients with post‐COVID‐19 syndrome.

**Methods:**

One hundred twenty older patients with post‐COVID‐19 syndrome were randomly assigned to one of two groups: patients who carried out the telerehabilitation programme (*n* = 60; age: 65.0 ± 5.2; female: 14.2%) and a control group (*n* = 60; age: 64.3 ± 5.0; female: 24.5%). An incremental cardiopulmonary exercise testing, isokinetic strength test, and bioelectrical impedance analysis were performed to compare cardiorespiratory and muscle strength responses and body composition between telerehabilitation and control groups.

**Results:**

A significant increase in the cardiopulmonary exercise testing duration was found in the telerehabilitation group compared to the control group (mean difference = 88.9 s, *P* = 0.001). Peak oxygen uptake increased in the telerehabilitation group (mean difference = 3.0 mL·kg^−1^·min^−1^, *P* < 0.001) and control group (mean difference = 1.9 mL·kg^−1^·min^−1^, *P* < 0.001). Power output in cycle ergometer (mean difference = 25.9 watts, *P* < 0.001), fat free mass (mean difference = 2.1 kg, *P* = 0.004), soft lean mass (mean difference = 2.1 kg, *P* = 0.003), and skeletal muscle mass (mean difference = 1.4 kg, *P* = 0.003) only increased in the telerehabilitation group. A significant increase in the power output was observed in the telerehabilitation group compared with the control group in both lower limbs after isokinetic strength test of the leg extension at a speed of 60° (right: mean difference = 18.7 watts, *P* = 0.012; left: mean difference = 15.3 watts, *P* = 0.010). The peak torque of right leg extension increased only in the telerehabilitation group after isokinetic strength test at a speed of 60° (mean difference = 13.1 N·m, *P* < 0.001). A significant increase in the power output was observed in the telerehabilitation group compared with the control group in the left leg extension after isokinetic strength test at a speed of 180° (mean difference = 30.2 watts, *P* = 0.003).

**Conclusions:**

The telerehabilitation programme improved cardiorespiratory and muscular fitness, and body composition in older patients with post‐COVID‐19 syndrome to a greater extent than a control group. The telerehabilitation programmes may be an alternative to improve the sequelae of post‐COVID‐19 syndrome in older patients.

## Introduction

Post‐coronavirus‐19 (post‐COVID‐19) syndrome or long COVID‐19 is defined as the presence of continuous acute symptoms after COVID‐19 infection; although the precise definition or duration of post‐COVID‐19 syndrome is still unclear, current research considers this syndrome to be a multisystem disease.^1^ The National Institute for Health and Care Excellence guidelines suggested a three‐month period to define post‐COVID‐19 syndrome.^2^

The main sequelae of post‐COVID‐19 syndrome are associated with functional (activities of daily living) and physical deterioration (difficulty breathing, muscle weakness and pain, fatigue), psychological alterations (anxiety and depression), mental disorders (cognitive impairment), and impaired pulmonary and muscular function during exercise.^1^, ^2^, ^3^, ^4^, ^5^ In this sense, it has been suggested that older people with COVID‐19 are very susceptible to post‐COVID‐19 syndrome,^1^, ^4^ rapidly decreasing their quality of life and increasing the risk of long‐term morbidity.^4^, ^6^

The post‐COVID‐19 syndrome can be accentuated by a prolonged stay in the hospital and especially in the intensive care unit (ICU).^7^ Prolonged hospitalization can impair physical function increasing long‐term muscle weakness, fatigue and decreasing muscle mass and exercise capacity even up to 6 months after acute COVID‐19 infection.^7^, ^8^, ^9^ As a result of bed rest, muscle mass and muscle strength may be reduced in older people.^10^

Scientific literature has emphasized the importance of rehabilitation and exercise programmes to improve the symptoms of post‐COVID‐19 syndrome.^11^ In addition, telerehabilitation programmes have proven to be a safe, viable, and effective alternative with high adherence for COVID‐19 recovery.^12^ Telerehabilitation conducted in the patient's home could offer a viable solution to aid patients in recovering from post‐COVID‐19 sequelae. Following the acute onset of COVID‐19 symptoms, clinical manifestations and complications may persist for over 12 weeks without being attributed to an alternative diagnosis.^2^ Hospitalization is unnecessary for the majority of patients. Telerehabilitation programmes at the patient's home supervised by therapists could provide a highly suitable alternative for enhancing cardiorespiratory and muscular fitness in patients,^13^ particularly older people, while reducing the risk of potential reinfection or virus transmission.^14^

Studies in older patients with COVID‐19 who have completed a rehabilitation or exercise programme are scarce. These studies primarily focus on the subacute, acute, and early recovery phases of COVID‐19 during and immediately after hospitalization.^15^ A comprehensive rehabilitation treatment in the subacute phase has been shown to be suitable for earlier recovery and to reduce the length of stay of patients in acute care.^16^ In addition, respiratory rehabilitation has also improved respiratory function assessed by spirometry, exercise capacity during the 6‐minute walk test, quality of life, and anxiety in older patients with COVID‐19.^17^

Multidisciplinary rehabilitation programmes appear to be an indispensable alternative in the acute, subacute and early recovery phases of COVID‐19 in older patients, especially during prolonged hospital and ICU stays.^15^ Despite the importance of rehabilitation in the early phases of recovery in older patients with COVID‐19, the effects of rehabilitation programmes in older people with long COVID‐19 have not received the same attention in the field of research.

Evaluation of cardiorespiratory fitness by the measurement of peak oxygen uptake (*V̇*O_2peak_), exercise capacity, and ventilatory efficiency using a cardiopulmonary exercise testing (CPET) is essential to predict morbidity and mortality in cardiorespiratory diseases and older people.^18^, ^19^ The evaluation of muscular fitness by measuring muscle strength and body composition is fundamental to determine functional limitations,^20^ and cardiometabolic and mortality risk factors.^21^, ^22^ In addition, rehabilitation programmes are essential for prompt recovery of cardiorespiratory and muscular fitness in adult patients with post‐COVID‐19 sequelae^13^; however, the effects of a rehabilitation programme in older people with post‐COVID‐19 syndrome on cardiorespiratory and muscular fitness, and body composition have not been explored.

This study aimed to assess the effects of a telerehabilitation programme on cardiorespiratory and muscular fitness, and body composition in older people with post‐COVID‐19 syndrome. Given the positive effects of exercise and rehabilitation programmes in the acute, subacute and early recovery phases of COVID‐19 in older patients, and in adult patients with post‐COVID‐19 syndrome, we hypothesized that a telerehabilitation programme improves exercise capacity, cardiorespiratory and muscular fitness, and body composition in older people with post‐COVID‐19 syndrome.

## Methods

### Study design

The methods for this study were based on a previously published research project.^23^ The present randomized parallel‐controlled trial was performed from 1 January 2023 to 31 January 2024 and was carried out with concealed allocation, blinded assessment of outcomes, and intention‐to‐treat analysis.

A total of 120 volunteer older patients with post‐COVID‐19 syndrome were recruited from the hospital and randomly assigned by simple computerized randomization to two study groups: patients who underwent the telerehabilitation programme (TRP group, *n* = 60) and a control group (CG, *n* = 60). Outcome measures were collected in both groups before (pretest) and after (post‐test) the telerehabilitation programme. A single researcher performed the allocation sequence and then assigned the participants to one of the study groups. This researcher did not participate at any time in the intervention and assessment process. The therapist who carried out the rehabilitation programme and the participants were not blinded to allocation. Other researchers carried out the pre‐ and post‐test evaluations before and after the intervention programme and were blinded to the two study groups.

The study was performed in accordance with the Recommendations for Interventional Trials,^24^ and was approved the by the Ethical Committee of Investigation of the Health Care Consortium (CEIm, Code: 16/21). Eligible patients gave informed consent before undergoing baseline evaluation and being allocated to one of the two study groups. This study was established according to the guidelines for reporting parallel group randomized trials of the CONSORT Statement.^25^

### Participants

The study sample was composed of patients recruited from the hospital with a clinical and functional diagnosis of COVID‐19. Adjusted morbidity groups were obtained from all participants.^26^ Data related to hospital admission were collected and the Acute Physiology and Chronic Health Disease Classification System II (APACHE II) was used to predict disease severity.^27^ The main symptoms of patients with post‐COVID‐19 syndrome were dyspnoea, persistent fatigue, and muscle weakness.

The inclusion criteria were age > 60 years, molecular diagnosis (reverse transcription polymerase chain reaction) of infection by SARS‐CoV‐2 > 3 months before randomization and suffering from post‐COVID sequelae >3 months from the onset of symptoms. The exclusion criteria included the absence of signed informed consent, severe neurological disease, active oncological disease, neuromuscular disease, orthopaedic disorders, or any disease or disorder that prevented the patient with post‐COVID‐19 syndrome to perform the intervention and evaluation process.

The relative *V̇*O_2peak_ was used as the primary variable to calculate sample size. Sample size was calculated using alpha <0.05 (5% probability of a type I error), providing a power of 80% (1‐β) using a two‐tailed alpha test and considering other prognostic covariates. A 2.6 mL·kg^−1^·min^−1^ higher mean difference in the TRP group compared with the CG was expected. As in a previous study, a similar sample of 45 patients per group was established to detect statistically significant differences between the two study groups.^13^, ^28^

The Department of Physical Medicine and Rehabilitation of the Mataró hospital established the guidelines of the rehabilitation programme, which was carried out by an experienced physiotherapist in therapeutic exercise for chronic diseases.

### Rehabilitation programme

The rehabilitation programme guidelines were previously described (Table 1).^13^, ^23^ Briefly, each patient engaged in the programme from the comfort of their own home, while the physiotherapist oversaw their exercise routines remotely via a camera installed in the hospital rehabilitation room. The platform used was Zoom (Video Communications, Inc., San José, California). Supervision of exercise at home was provided by a family member. To enhance motivation, sessions were conducted in group settings with patients connected via the network. The physiotherapist incorporated gaming elements into the sessions as part of the telerehabilitation programme strategy. Additionally, the rehabilitation doctor conducted regular individualized consultations at the hospital to monitor the patient's progress and adjust the telerehabilitation programme based on each patient's improvement.^29^ The family member had the physiotherapist's telephone number to notify of any setback during the telerehabilitation programme. Rehabilitation sessions were suspended in case of adverse events (e.g., dizziness and mild lipothymia).

**Table 1:** Characteristics of the rehabilitation programme

Session	Methodology	Frequency	Duration	Exercises	Set	Rep/T	Rest E/S	Intensity
Warm‐up	General/JM	3 sessions/w	10 min	1–3	1	5–15/20 s	15/60–120 s	HR: 40–50% RPE: 4–5
Workout	CT	3 sessions/w	50 min	8–12	2–4	5–25/60 s	15/60–120 s	HR to VT1: 50–75% RPE: 4–8
Cooling down	Stretching/relaxation	3 sessions/w	10 min	1–8	1	20–30 s	15/60–120 s	Resting HR RPE: 1–3	

CT, circuit training; E/S, exercise/set in seconds; HR, heart rate; JM, joint mobility; Rep/T, Repetitions/effort time in seconds; RPE, ratio of perceived exertion; VT1, first ventilatory threshold; w, weekly.

Older participants with post‐COVID‐19 syndrome completed a total of 12 weeks (3 weekly sessions) of a telerehabilitation programme with aerobic and strength exercises using a circuit training methodology. Exercise intensity was controlled by the heart rate obtained at the first ventilatory threshold (VT1) during the CPET and the modified BORG scale (CR‐10).^13^, ^30^, ^31^ The exercises of the rehabilitation programme were steps (aerobic), upper and lower extremity and CORE exercises, such as knee elevations, elbow, shoulder, knee, ankle and neck extension and flexion, abduction‐adduction of shoulders, hips, squats, jumps, scissors, and callisthenics exercises.

Older participants with post‐COVID‐19 syndrome showed adequate adherence to the rehabilitation programme by completing at least 80% of the scheduled sessions.^13^ Adherence to the telerehabilitation programme was monitored by the physical therapist by quantifying participant dropout, attendance, and active participation in the sessions. A dropout was defined as a participant who did not participate in the intervention as required by the study protocol due to illness, death, or voluntary withdrawal from the rehabilitation programme. Adherence was operationalized as the degree to which participants complied with the telerehabilitation programme sessions.^30^

### Control group

Older participants with post‐COVID‐19 syndrome of the CG did not perform the telerehabilitation programme. They carried out their routine activities of daily living.

In both experimental groups, participants who were unable to perform activities of daily living independently were excluded from the study.

### Outcome measures

An intention‐to‐treat analysis was used. All registered primary and secondary outcomes of the TRP group and the CG were considered and reported in the final data analysis.^32^

Primary outcome measures were based on the assessment of cardiorespiratory and muscular fitness, and body composition in older patients with post‐COVID‐19 syndrome. The evaluations (pre‐ and post‐test) were carried out in the Department of Physical Medicine and Rehabilitation of the Mataró hospital.

Cardiorespiratory fitness was assessed by incremental CPET until voluntary fatigue. The CPET was supervised and performed by the cardiac rehabilitation unit and was stopped in case of abnormal blood pressure or electrocardiographic alterations. Participants performed the same CPET before (pre‐test) and after the rehabilitation programme (post‐test). The CPET was carried out on an ergometric bicycle with electromagnetic brakes (Ergoline900S, Ergoline GmbH, Bitz, Germany). The exercise protocol was individualized and adapted to the physical condition of each patient with increases of 5 W·min^−1^. The position on the cycle ergometer was adjusted for each participant. The participants, seated on the bicycle ergometer with their corresponding mask, were asked to maintain a constant pedalling cadence of between 50 and 70 revolutions per minute in the CPET.^13^

Pulmonary gas exchange data were collected during CPET using an open‐circuit breathing gas analyser (Ergostik, Geratherm Respiratory, Bad Kissingen, Germany) calibrated before each test. Patients were monitored by continuous 12‐lead electrocardiography and blood pressure was measured. Data on relative and absolute *V̇*O_2peak_, minute ventilation (VE), ventilatory equivalent for oxygen (VE·*V̇*O_2_^−1^), ventilatory equivalent for carbon dioxide (VE·*V̇*CO_2_^−1^), respiratory exchange ratio (RER), and end‐tidal partial pressure of oxygen and carbon dioxide (PetO_2_ and PetCO_2_, respectively) were obtained. The VT1 was established as in a previous study.^13^

Muscular fitness of the lower limbs was evaluated using an isokinetic strength test. The isokinetic knee flexor and extensor testing was carried out in a concentric‐concentric regime at low (60° per second) and high speeds (180° per second) using the Biodex Advantage system (Software V.4X; Biodex, Shirley, NY, USA). The variable analysed in the study was the maximum moment of force of extension and flexion of the knee at low and high speeds, with the highest value of the different repetitions recorded.^33^ The isokinetic knee flexor and extensor testing protocol included (1) training, consisting of an 80° range of mobility valuation, 5 repetitions of 60° per second, 20‐s rest, five repetitions of 180° per second‐ and 15‐s rest. (2) Test consisting of two series of five repetitions of 60° per second concentric‐concentric regime, alternating with two series of five repetitions of 180° per second concentric‐concentric regime, with all the series being separated by resting periods of 60 s.

Body composition was assessed by bioelectrical impedance analysis (BIA), which provides estimates of fat mass (FM), fat free mass (FFM), and muscle mass (MM) in kg and as a percentage of body weight, total body water (TBW) in litres and as a percentage of bodyweight, and extracellular (ECW) and intracellular water (ICW) in litres and as a percentage of TBW. The InBody S10® multi‐frequency system (Biospace, California, USA) was used in the following standard conditions: no intense exercise in the previous 24 h, no alcohol consumption in the previous 8 h, strict fasting in the previous 2 h, and a toilet visit prior to the evaluation; this BIA device applies 1, 5, 50, 250, 500, and 1000 Hz frequencies. The following variables were assessed: body mass in kg, body FM (BFM) in kg and percentage (%), FFM in kg, soft lean mass (SLM) in kg and skeletal muscle mass (SMM) in kg.

Secondary outcomes were based on spirometry test evaluation. Standard spirometry testing was performed using the same Ergostik cardiopulmonary exercise system combined with Blue Cherry diagnostic software. The following spirometry parameters were analysed: forced vital capacity (FVC), forced expiratory volume in 1 s (FEV1). For each patient, three comparable tests were performed with sufficient compliance and satisfactory data quality.

### Data analysis

The SPSS software version 25.0 for Mac (SPSS Inc., Chicago, IL, USA.) was used to perform the statistical analyses. The evaluation of data distribution was carried out with the Kolmogorov–Smirnov test and the data were presented as means and standard deviation and means and their 95% confidence interval (95% CI). Logarithmic transformation of data on cardiorespiratory, muscular and body composition variables with non‐normal distribution was performed. The homogeneity of the variance was analysed with the Levene test. A general linear model of mixed repeated measures (ANOVA) adjusted for age and sex was applied. A within‐group factor (time effect) in two levels (pre‐ and post‐test) and an inter‐group factor (intervention effect) (TRP and CG) were applied. The effect of interaction (intervention•time) was analysed. The Greenhouse–Geisser correction was used when the Mauchly W led to rejection of the sphericity test. When appropriate, Bonferroni adjustment was applied to identify multiple comparisons between the experimental groups. The magnitude of response in the different groups was estimated with partial eta‐squared (*η*_p_^2^). The scale of *η*_p_^2^ classification was small (*η*_p_^2^ = 0.01), moderate (*η*_p_^2^ = 0.06) and large (*η*_p_^2^ = 0.14).^34^ The statistical power (SP) was also calculated.

In the descriptive and clinical data of patients with post‐COVID‐19 syndrome, the Chi‐square statistic test, the Student's *t*‐test for independent samples or the Mann–Whitney test were used to verify significant differences between the study groups. Statistical significance was set at *P* < 0.05.

## Results

### Participants

**Figure 1:**
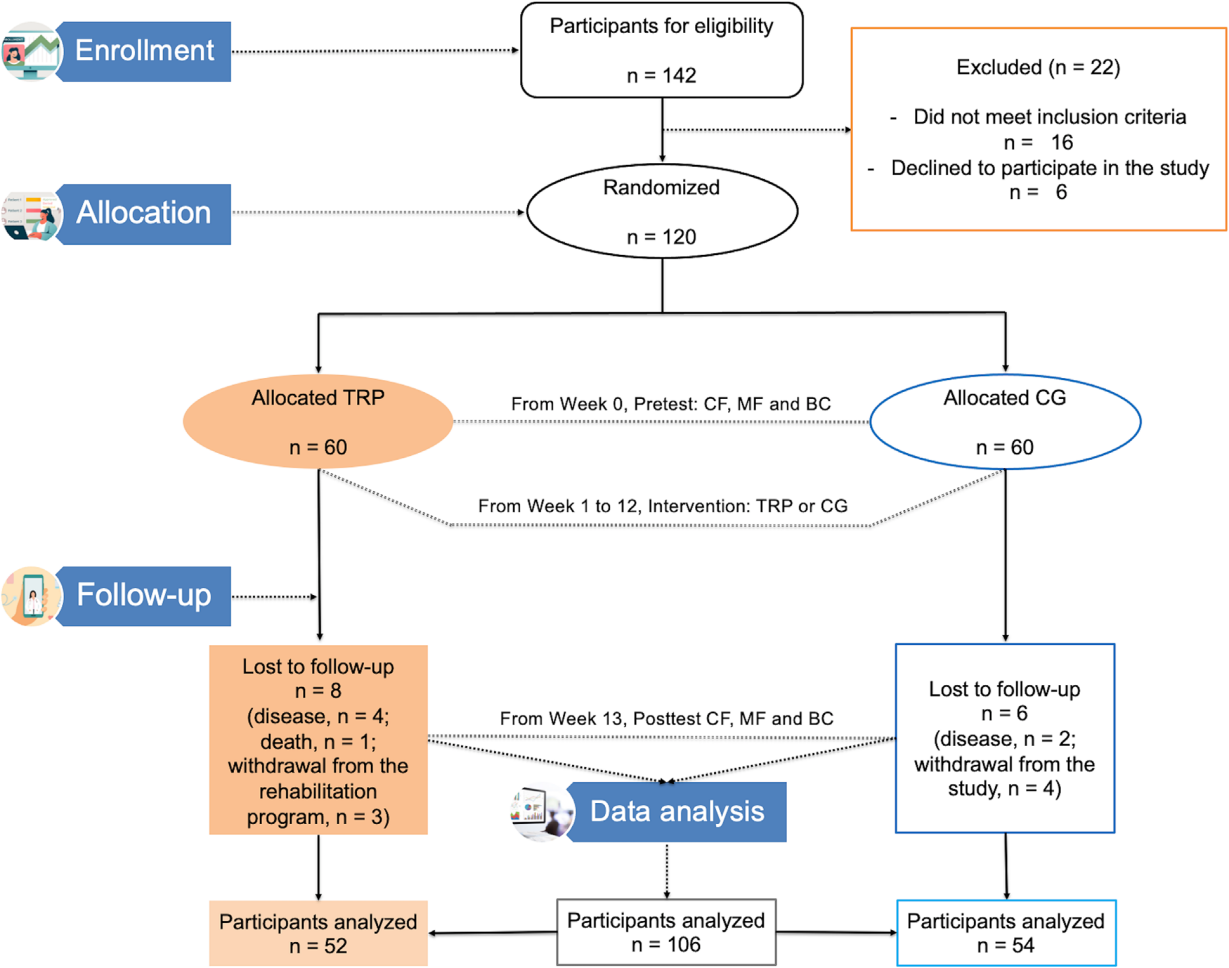
CONSORT flowchart. BC, body composition; CF, cardiorespiratory fitness; CG, control group; MF, muscular fitness; TRP, telerehabilitation programme group.

Figure 1 shows the enrolment, allocation, follow‐up, and data analysis of older patients with post‐COVID‐19 sequelae. One hundred seventy older patients with post‐COVID‐19 sequelae were initially contacted. Of these, 142 met the inclusion criteria and 120 were randomly assigned to TRP (*n* = 60) or CG (*n* = 60). One hundred six older patients with post‐COVID‐19 sequelae completed the rehabilitation programme and the evaluations of cardiorespiratory and muscular fitness and body composition (TRP, *n* = 52; CG, *n* = 54). Of the 52 older patients with post‐COVID‐19 syndrome who followed the rehabilitation programme, 49 met the established adherence criteria of 80%.

The descriptive and clinical data of the older patients with post‐COVID‐19 syndrome are shown in Table 2. Those in the TRP group showed higher hospitalizations (chi‐square = 4.9, *P* = 0.027), more days hospitalized (*t* = 2.7, *P* = 0.008), ICU admissions (chi‐square = 5.7, *P* = 0.017), mechanical ventilation (chi‐square = 11.1, *P* = 0.001), tracheostomy (chi‐square = 6.6, *P* = 0.010), and prone position (chi‐square = 7.7, *P* = 0.005) than those in the GC.

**Table 2:** Descriptive and clinical data of older patients with post‐COVID‐19 syndrome

Variable	TRP	CG
Female (*n*, %)	F (*n* = 15/14.2%)	F (*n* = 26/24.5%)
Male (*n*, %)	M (*n* = 37/34.9.0%)^¶^	M (*n* = 28/26.4%)
Age (years)	65.0 (5.2)	64.3 (5.0)
Height (m)	1.7 (0.1)	1.6 (0.1)
Weight (kg)	79.6 (17.5)	82.3 (17.6)
BMI (kg•m^−2^)	28.7 (5.2)	30.3 (5.7)
Hospitalization, yes (%)	45 (86.5%)*	37 (68.5%)
Hospital admission, days	29.8 (26.4)*	16.1 (20.9)
ICU admission, yes (%)	24 (46.2%)*	13 (24.1%)
ICU admission, days	23.7 (20.0)	20.0 (17.4)
Dyspnoea, yes (%)	38 (73.1%)	39 (72.2%)
MW and/or fatigue, yes (%)	51 (98.1%)	54 (100%)
Co‐morbidities, yes (%)	36 (69.2%)	36 (66.7%)
Charlson index	2.7 (1.4); 0 = 3.8%; ≤ 3 = 48.1%	2.3 (1.2); 0 = 0%; ≤ 3 = 81.5%
Mechanic ventilation, yes (%)	12 (23.1%)*	1 (1.9%)
Tracheostomy, yes (%)	6 (11.5%)*	0 (0%)
Prone position, yes, (%)	16 (30.8%)*	5 (9.3%)

Data are provided as mean (standard deviation or percentage).

BMI, body mass index; CG, control group; F, female; ICU, intensive care unit; M, male; MW, muscle weakness; n, number of patients with post‐COVID‐19 syndrome; TRP, telerehabilitation group.

* Significantly higher than CG (*P* < 0.001).

^¶^ Significantly higher than female patients in TRP (*P* = 0.020). *n* = 106, telerehabilitation *n* = 52; control group *n* = 54.

### Primary outcomes

Older patients complied with randomly assigned interventions and underwent all the planned assessments. The researchers encouraged continued participation in scheduled assessments when a participant was unable to complete any of the planned tests. All the data obtained from older patients with post‐COVID‐19 syndrome were included in the statistical analysis maintaining the originally randomized study group.

**Figure 2:**
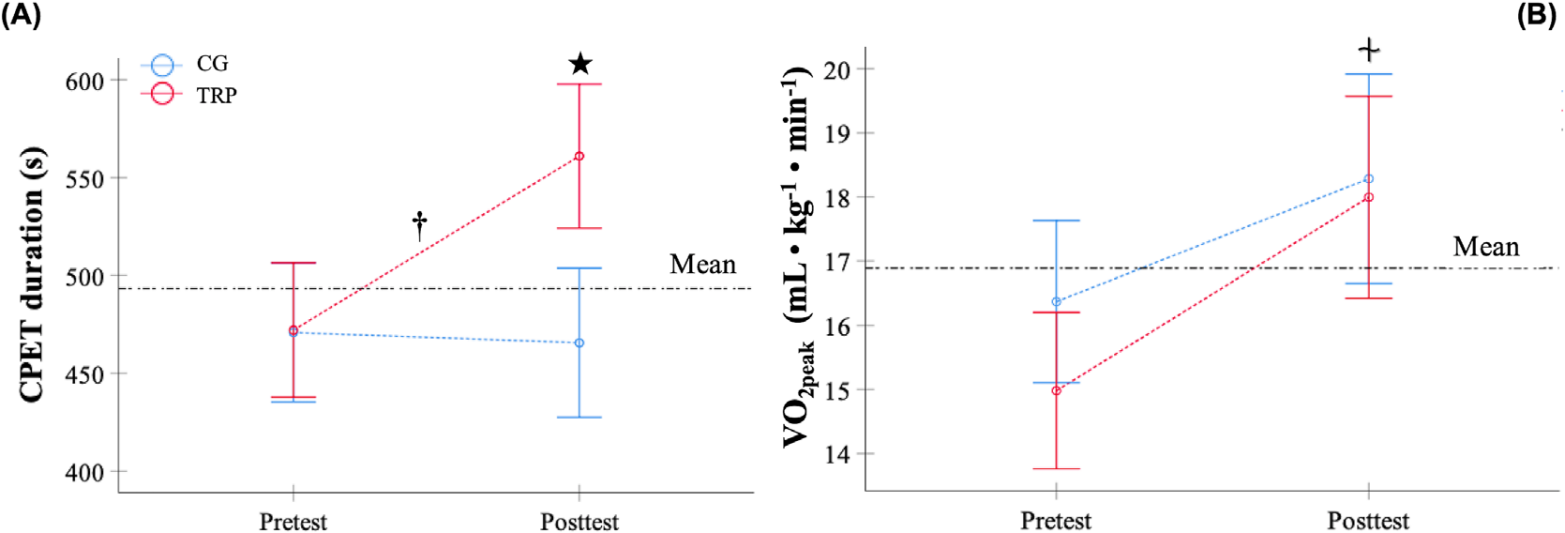
Differences in cardiopulmonary exercise test duration (A) and peak oxygen uptake (B) between the telerehabilitation programme and the control group. CG, control group; CPET, cardiopulmonary exercise test; TRP, supervised telerehabilitation programme; *V̇*O_2peak_, peak oxygen uptake. †Significant increase in the TRP group compared with CG after the telerehabilitation programme (*P* = 0.001). ★Significant increase in the TRP group after the telerehabilitation programme (*P* < 0.001). ⏆Significant increase in both study groups after the telerehabilitation programme (*P* < 0.001).

The cardiorespiratory fitness results during the CPET are shown in Table 3 and Figure 2.

**Table 3:** Changes in cardiorespiratory fitness (CPET) and body composition (BIA) after the telerehabilitation programme.

Outcome measures	EG	Within‐group (change from pre‐ to post‐test)		Time	Intergroup (difference from pre‐ to post‐test)	
		Mean diff. (95% CI)	*P*‐value		Mean diff. (95% CI)	*P*‐value
CPET		Pre − Post			CG − TRP	
VE·VCO_2_^−1^ slope*	CG	0.0 (−0.0 to 0.0)	0.814	Pre	−0.1 (−0.1 to 0.0)	0.006
	TRP	0.0 (−0.0 to 0.0)	0.219	Post	−0.0 (−0.1 to 0.0)	0.041
Peak HR (beats·min^−1^)	CG	−0.0 (−3.3 to 3.3)	0.990	Pre	7 (−0.2 to 14.2)	0.057
	TRP	−6.3 (−9.5 to −3.2)	<0.001	Post	0.7 (−7.2 to 8.6)	0.863
Peak VE (L·min^−1^)	CG	−8.3 (−11.8 to −4.8)	<0.001	Pre	3.4 (−2.3 to 9.2)	0.238
	TRP	−9.8 (−13.2 to −6.4)	<0.001	Post	2.0 (−4.7 to 8.6)	0.564
Peak RER	CG	−0.1 (−0.2 to 0.0)	0.075	Pre	0.1 (−0.0 to 0.2)	0.110
	TRP	−0.2 (−0.3 to −0.1)	<0.001	Post	−0.0 (−0.1 to 0.1)	0.527
Power output (W)	CG	−5.8 (−11.8 to 0.3)	0.061	Pre	21.0 (9.7 to 32.3)	<0.001
	TRP	−25.9 (−31.7 to −20.0)	<0.001	Post	0.9 (−13.0 to 14.8)	0.898
Saturation (%)	CG	−0.0 (−0.0 to 0.0)	0.207	Pre	0.0 (−0.0 to 0.0)	0.136
	TRP	−0.0 (−0.0 to −0.0)	0.013	Post	0.0 (−0.0 to 0.0)	0.237
Body composition (BIA)						
Body mass (kg)	CG	−0.4 (−1.5 to 0.7)	0.457	Pre	4.4 (−2.3 to 11.0)	0.196
	TRP	−1.9 (−3.0 to −0.8)	0.001	Post	2.9 (−3.8 to 9.5)	0.393
Body fat mass (kg)	CG	−0.3 (−1.6 to 0.9)	0.570	Pre	−2.6 (−8.3 to 3.1)	0.370
	TRP	0.8 (−0.6 to 2.1)	0.254	Post	−1.5 (−7.2 to 4.2)	0.608
Body fat (%)	CG	−0.1 (−1.5 to 1.3)	0.894	Pre	−1.4 (−5.3 to 2.5)	0.479
	TRP	1.5 (−0.1 to 3.0)	0.063	Post	0.2 (−4.0 to 4.3)	0.936
Fat free mass (kg)	CG	−0.0 (−1.3 to 1.3)	0.974	Pre	0.5 (−2.9 to 3.8)	0.783
	TRP	−2.1 (−3.6 to −0.7)	0.004	Post	−1.7 (−4.9 to 1.5)	0.305
Soft lean mass (kg)	CG	0.0 (−1.2 to 1.3)	0.986	Pre	0.5 (−2.7 to 3.6)	0.760
	TRP	−2.1 (−3.4 to −0.7)	0.003	Post	−1.6 (−4.6 to 1.4)	0.295
Skeletal muscle mass (kg)	CG	−0.0 (−0.9 to 0.8)	0.930	Pre	0.3 (−1.7 to 2.2)	0.788
	TRP	−1.4 (−2.3 to −0.5)	0.003	Post	−1.1 (−3.0 to 0.8)	0.256

Data are provided as mean difference and 95% confidence intervals (95% CI).

BIA, bioelectrical impedance analysis; CG, control group; CPET, cardiopulmonary exercise test; Diff., difference; EG, experimental group; HR, heart rate; Post, test after telerehabilitation programme; Pre, test before telerehabilitation programme; RER, respiratory exchange ratio; TRP, telerehabilitation programme; VCO_2_, carbon dioxide production; VE, ventilation.

* Logarithmic transformation of data.

In relation to exercise capacity (CPET duration), an intervention•time interaction effect was detected (*P* = 0.001, *F* = 15.4, *η*_p_^2^ = 0.2, SP = 1.0). Bonferroni adjustment showed a significant increase in the CPET duration in the TRP group compared with the CG (*P* = 0.001). Only the TRP group improved the duration of CPET (*P* < 0.001) (Figure 2A).

In *V̇*O_2peak_, an intervention•time interaction effect was verified (*P* = 0.05, *F* = 3.9, *η*_p_^2^ = 0.0, SP = 0.5). Bonferroni adjustment verified a significant increase in *V̇*O_2peak_ in both groups (*P* < 0.001). No significant changes were observed between the two groups (*P* > 0.05) (Figure 2B).

An intervention•time interaction effect was identified in the power output (*P* < 0.001, *F* = 22.0, *η*_p_^2^ = 0.2, SP = 1.0). Bonferroni adjustment indicated that only the TRP group increased the power output after the rehabilitation programme (*P* < 0.001).

In regard to heart rate, an intervention•time interaction effect was identified (*P* = 0.008, *F* = 7.3, *η*_p_^2^ = 0.1, SP = 0.8). Bonferroni adjustment showed that the heart rate increased in the TRP group after the rehabilitation programme (*P* < 0.001).

No intervention•time interaction effect was found in the other cardiorespiratory fitness variables (*P* > 0.05).

**Figure 3:**
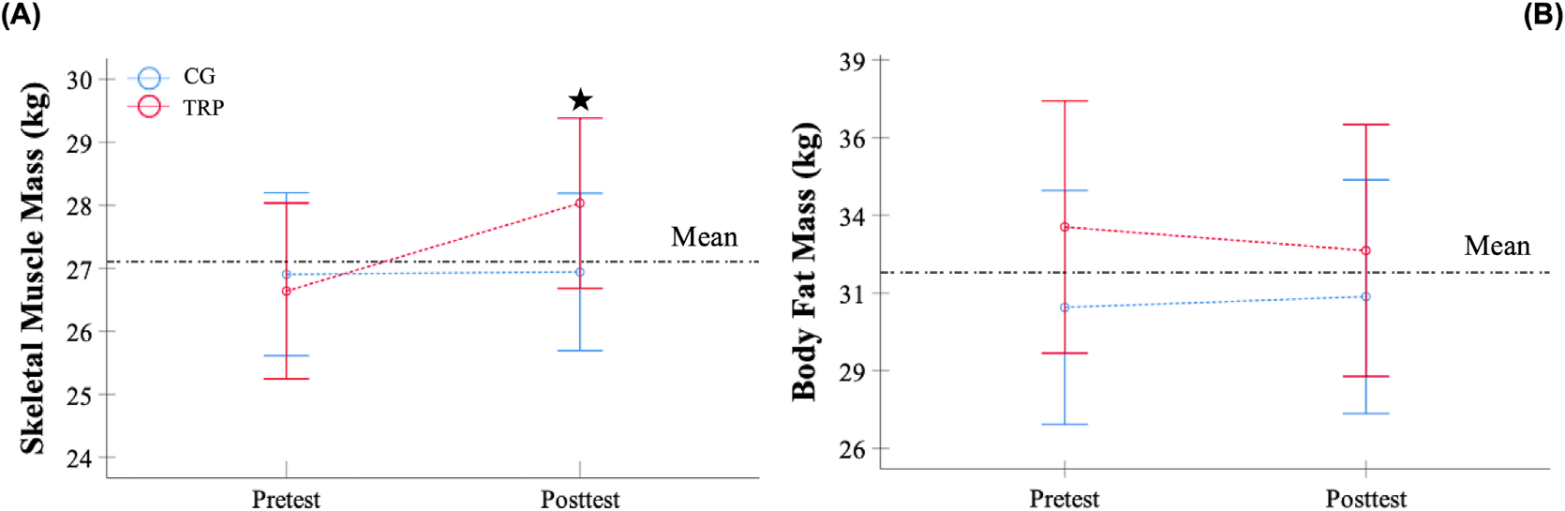
Differences in skeletal muscle mass (A) and body fat mass (B) between the telerehabilitation programme group and the control group. CG, control group; TRP, telerehabilitation programme group. ★Significant increase in the TRP group after the telerehabilitation programme (*P* = 0.003).

The body composition results during the BIA assessment are shown in Table 3 and Figure 3.

An intervention•time interaction effect was found in FFM, SLM and SMM (*P* = 0.039, *F* = 4.4, *η*_p_^2^ = 0.1, SP = 0.5; *P* = 0.032, *F* = 4.8, *η*_p_^2^ = 0.1, SP = 0.6; *P* = 0.034, *F* = 4.7, *η*_p_^2^ = 0.1, SP = 0.6, respectively). Bonferroni adjustment determined that FFM, SLM and SMM only increased in the TRP group (Figure 3A) (*P* ≤ 0.004).

No intervention•time interaction effect was identified in body mass and BFM (Figure 3B) and BF (%) (*P* > 0.05).

Muscle strength results during the isokinetic test are shown in Table 4. In the peak torque of leg extension at a speed of 60°, an intervention•time interaction effect was identified in right leg extension (*P* = 0.019, *F* = 5.8, *η*_p_^2^ = 0.1, SP = 0.6). Bonferroni adjustment showed that the peak torque of right leg extension increased only in the TRP group after the rehabilitation programme (*P* < 0.001). No intervention•time interaction effect was identified in left leg extension (*P* = 0.060), although the peak torque of left leg extension increased only in the TRP group following the rehabilitation programme (*P* = 0.001).

**Table 4:** Changes in isokinetic strength testing after the telerehabilitation programme

Outcome measures	EG	Within‐group (change from pre‐ to post‐test)		Time	Intergroup (difference from pre‐ to post‐test)	
		Mean diff. (95% CI)	*P*‐value		Mean diff. (95% CI)	*P*‐value
Peak torque (N·m)		Pre–Post			CG–TRP	
60° right knee ext.	CG	−4.5 (−9.7 to 0.73)	0.090	Pre	−6.4 (−24.3 to 11.6)	0.482
	TRP	−13.1 (−17.6 to −8.6)	<0.001	Post	−15.0 (−36.2 to 6.2)	0.163
60° left knee ext.	CG	−1.1 (−8.9 to 6.6)	0.774	Pre	0.3 (−15.3 to 15.9)	0.970
	TRP	−11.3 (−18.1 to −4.6)	0.001	Post	−9.9 (−28.5 to 8.7)	0.291
180° right knee ext.	CG	−0.0 (−0.1 to 0.0)	0.270	Pre	−0.1 (−0.2 to 0.0)	0.105
	TRP	−0.0 (−0.1 to 0.0)	0.123	Post	−0.1 (−0.2 to 0.0)	0.164
180° left knee ext.	CG	3.2 (−5.4 to 11.7)	0.463	Pre	−11.5 (−22.3 to −0.6)	0.039
	TRP	−6.7 (−14.1 to 0.8)	0.078	Post	−21.3 (−36.4 to −6.2)	0.007
60° right knee flex.	CG	−8.4 (−13.7 to −3.1)	0.002	Pre	−11.5 (−22.3 to −0.7)	0.038
	TRP	−4.1 (−8.8 to 0.7)	0.092	Post	−7.2 (−21.6 to 7.2)	0.324
60° left knee flex.	CG	−4.0 (−9.1 to 1.1)	0.123	Pre	−9.4 (−19.7 to 1.0)	0.074
	TRP	−1.8 (−6.4 to 2.8)	0.432	Post	−7.2 (−19.5 to 5.1)	0.248
180° right knee flex.*	CG	−0.0 (−0.1 to 0.0)	0.270	Pre	−0.1 (−0.2 to 0.0)	0.105
	TRP	−0.0 (−0.1 to 0.0)	0.123	Post	−0.1 (−0.2 to 0.0)	0.164
180° left knee flex.	CG	1.8 (−4.0 to 7.5)	0.538	Pre	−17.2 (−25.9 to −8.6)	<0.001
	TRP	−0‐0 (−4.8 to 4.7)	0.985	Post	−19.1 (−30.1 to −8.0)	0.001
Power output (W)						
60° right knee ext.	CG	−3.9 (−9.3 to 1.4)	0.143	Pre	−6.8 (−17.3 to 3.7)	0.198
	TRP	−15.8 (−20.5 to −11.2)	<0.001	Post	−18.7 (−33.2 to −4.2)	0.012
60° Left knee ext.	CG	0.6 (−4.4 to 5.5)	0.825	Pre	−5.0 (−14.9 to 5.0)	0.322
	TRP	−9.8 (−14.1 to −5.4)	<0.001	Post	−15.3 (−26.8 to −3.8)	0.010
180° right knee ext.	CG	−0.1 (−0.2 to 0.0)	0.040	Pre	−0.1 (−0.2 to 0.0)	0.042
	TRP	−0.1 (−0.2 to 0.0)	0.018	Post	−0.1 (−0.3 to 0.0)	0.083
180° left knee ext.	CG	0.1 (−13.1 to 13.3)	0.987	Pre	−8.3 (−19.9 to 3.3)	0.159
	TRP	−21.8 (−33.2 to −10.3)	<0.001	Post	−30.2 (−49.9 to −10.4)	0.003
60° right knee flex.	CG	−5.8 (−9.4 to −2.3)	0.002	Pre	−13.0 (−21.2 to −4.8)	0.002
	TRP	−5.6 (−8.8 to −2.4)	0.001	Post	−12.8 (−23.3 to −2.3)	0.018
60° left knee flex.	CG	−2.6 (−6.0 to 0.8)	0.129	Pre	−7.8 (−14.9 to −0.6)	0.034
	TRP	−5.1 (−8.1 to −2.0)	0.001	Post	−10.2 (−18.9 to −1.5)	0.022
180° right knee flex.*	CG	−0.1 (−0.2 to −0.0)	0.040	Pre	−0.1 (−0.2 to −0.0)	0.042
	TRP	−0.1 (−0.2 to −0.0)	0.018	Post	−0.1 (−0.3 to 0.0)	0.083
180° left knee flex.	CG	−0.8 (−7.6 to 5.9)	0.803	Pre	−17.0 (−25.7 to −8.2)	<0.001
	TRP	−7.8 (−13.4 to −2.3)	0.007	Post	−23.9 (−36.0 to −11.9)	<0.001

Data are provided as mean difference and 95% confidence intervals (95% CI) between both experimental groups.

CG, control group; Diff., difference; EG, experimental group; Ext, extension; Flex, flexion; Post, test after telerehabilitation programme; Pre, test before telerehabilitation programme; TRP, telerehabilitation programme.

* Logarithmic transformation of data.

In the power output of the leg extension at a speed of 60°, an intervention•time interaction effect was observed in both lower limbs (right, *P* = 0.002, *F* = 10.7, *η*_p_^2^ = 0.1, SP = 0.9; left, *P* = 0.004, *F* = 9.2, *η*_p_^2^ = 0.1, SP = 0.8). Bonferroni adjustment showed a significant increase in the power output in both lower limbs in the TRP group compared with the CG (right, *P* = 0.012; left, *P* = 0.010). The power output in both lower limbs only improved in the TRP group (*P* < 0.001).

In the peak torque and power output of the leg flexion at a speed of 60°, no intervention•time interaction effect was observed in either lower limb (*P* > 0.05).

At a speed of 180°, only an intervention•time interaction effect was found in the power output of leg extension in the left lower limb (*P* = 0.018, *F* = 5.9, *η*_p_^2^ = 0.1, SP = 0.7). Bonferroni adjustment showed a significant increase in the power output in the left lower limb in the TRP group compared with the CG (*P* = 0.003). The power output in the left lower limb only improved in the TRP group (*P* < 0.001).

No intervention•time interaction effect was detected in either lower limb in the rest of the variables at a speed of 180° (*P* > 0.05).

### Secondary outcomes

No intervention•time interaction effect was found in the spirometry test variables (*P* > 0.05) (Table 5).

**Table 5:** Changes in spirometry test after the telerehabilitation programme

Outcome measures	EG	Withing‐group (change from pre‐ to post‐test)		Time	Intergroup (difference from pre‐ to post‐test)	
		Mean diff. (95% CI)	*P*‐value		Mean diff. (95% CI)	*P*‐value
Spirometry test		Pre–Post			CG–TRP	
FVC (L)*	CG	−0.0 (−0.0 to 0.0)	0.185	Pre	−0.0 (−0.0 to 0.1)	0.328
	TRP	−0.0 (−0.0 to −0.0)	0.021	Post	−0.0 (−0.0 to 0.1)	0.562
FVC (%)	CG	−6.4 (−11.3 to −1.4)	0.012	Pre	8.3 (0.3 to 16.2)	0.041
	TRP	−6.1 (−11.0 to −1.2)	0.015	Post	8.5 (1.1 to 16.0)	0.025
FEV1 (L)	CG	−0.1 (−0.2 to −0.0)	0.038	Pre	0.1 (−0.1 to 0.3)	0.408
	TRP	−0.2 (−0.3 to −0.1)	0.002	Post	0.0 (−0.2 to 0.3)	0.741
FEV1 (%)	CG	−3.6 (−7.2 to 0.1)	0.054	Pre	−0.8 (−8.2 to 6.6)	0.830
	TRP	−4.8 (−8.4 to −1.3)	0.009	Post	−2.1 (−10.2 to 6.0)	0.611
FEV1/FVC (%)*	CG	0.0 (−0.0 to 0.0)	0.958	Pre	0.0 (−0.0 to 0.0)	0.644
	TRP	0.0 (−0.0 to −0.0)	0.517	Post	0.0 (−0.0 to 0.0)	0.238

Data are provided as mean difference and 95% confidence intervals (95% CI).

CG, control group; Diff., difference; EG, experimental group; FEV1, forced expiratory volume in the first second; FVC, forced vital capacity; Post, test after telerehabilitation programme; Pre, Test before telerehabilitation programme; TRP, telerehabilitation programme.

* Logarithmic transformation of data.

## Discussion

The main finding of this study was that the telerehabilitation programme improved cardiorespiratory and muscular fitness, and body composition in older patients with post‐COVID‐19 syndrome to a greater extent than a control group.

The evaluation of *V̇*O_2peak_, exercise capacity, and ventilatory efficiency using a CPET has shown to be an essential procedure to determine cardiorespiratory fitness in adults with post‐COVID‐19 sequelae.^13^ In older patients with post‐COVID‐19 syndrome, exercise capacity and power output during a CPET in a cycle ergometer improved in the TRP group compared with the CG after the telerehabilitation programme. The *V̇*O_2peak_ increased in both groups; however, the rehabilitation programme was not more effective than activities of daily living (CG) to improve the *V̇*O_2peak_ and ventilatory efficiency in older people with post‐COVID‐19 syndrome.

Similar findings have been described in adult patients with post‐COVID‐19 sequelae. Exercise capacity, power output and mechanical efficiency were improved in subjects after a telerehabilitation programme compared with a control group.^13^ Studies evaluating the effects of a rehabilitation programme on cardiorespiratory fitness in older patients with COVID‐19 are scarce. To our knowledge, no study has evaluated the effects of a telerehabilitation programme on *V̇*O_2peak_ and exercise capacity during a CPET in older people with post‐COVID‐19 syndrome. However, a six‐week respiratory rehabilitation programme enhanced functional exercise capacity in older patients with COVID‐19.^17^

It has been suggested that rehabilitation interventions in adults with post‐COVID‐19 condition are associated with improvements in functional exercise capacity. However, the association of rehabilitation programmes with respiratory function is not clear enough.^35^ Several studies have verified no significant changes in *V̇*O_2peak_ or *V̇*O_2max_ between groups that performed an exercise programme compared with a CG. Patients with post‐COVID‐19 sequelae who completed an exercise or rehabilitation programme had similar *V̇*O_2peak_ or *V̇*O_2max_ increases after a 15‐week supervised telerehabilitation programme (~2.6 mL·kg^−1^·min^−1^),^13^ and an 8‐week concurrent training programme (~2.9 mL·kg^−1^·min^−1^) and concurrent training with inspiratory muscle training (~2.5 mL·kg^−1^·min^−1^).^28^ In these studies,^13^, ^28^ patients with long COVID‐19 did not recover the mean *V̇*O_2max_ lost (−4.9 mL·kg^−1^·min^−1^)^36^ after a rehabilitation and exercise programme.

Unlike our study, ventilatory efficiency improved in adults with post‐COVID‐19 sequelae after a rehabilitation programme.^13^ Perhaps, the intensity and type of aerobic exercise proposed in our rehabilitation programme was not intense and specific enough to recover *V̇*O_2peak_ and ventilatory efficiency in older people with post‐COVID‐19 sequelae.

Assessment of muscle strength and body composition is essential to determine functional limitations and cardiometabolic and mortality risk factors, especially in older adults.^20^, ^21^, ^22^ In this regard, the assessment of lower‐body muscle power output and strength is of great relevance due to its association with the ability to carry out activities of daily living.^37^ Leg extension muscular power output increased in the TRP group compared with the CG in both lower limbs (except right leg extension at 180° per second). In addition, leg extension peak torque only increased in the TRP group at low speed (60° per second) in both lower limbs. In adults with post‐COVID‐19 conditions, Jimeno‐Almazán *et al*. verified improvements in the lower body maximal and submaximal strength (half‐squat test) after concurrent training.^28^ A telerehabilitation programme was an essential tool to improve the extensor strength of the lower extremities in older patients with post‐COVID‐19 sequelae.

Interestingly, power output and peak torque leg flexion was not a differential factor observed in the intervention‐time interaction. Our rehabilitation programme probably did not have specific exercises to improve leg flexion power output and peak torque. The implementation of some specific leg flexion exercises would undoubtedly avoid some type of muscle decompensation (flexion/extension). In this regard, the assessment of leg flexion strength could be a better prognostic marker for mortality than leg extension strength in patients with post‐COVID‐19 sequelae, as occurs in chronic obstructive pulmonary disease.^38^ More studies are necessary to corroborate these claims in older people with post‐COVID‐19 syndrome.

In addition to the decrease in muscle strength, a loss of muscle mass is one of the main factors associated with fatigue, muscle weakness and sarcopenia in patients with post‐COVID‐19 sequelae.^39^ Fortunately, an increase in FFM, SLM, and SMM was observed in patients with post‐COVID‐19 syndrome who underwent the rehabilitation programme. This is a relevant and priority finding in research because, together with the gains observed in the strength of the lower limbs, sarcopenia and myopenia are being prevented in older people with post‐COVID‐19 sequelae with a greater tendency to lose muscle mass more quickly.^40^

However, this assumption should be taken with caution as no specific diagnosis of sarcopenia and myopenia was made in the study participants. The absence of a formal diagnosis of sarcopenia and myopenia could affect the precision of the conclusions and the generalization of the results.

Current research suggests that improvements in cardiorespiratory and muscular fitness, and body composition in older patients with post‐COVID‐19 syndrome could be strongly linked to the implementation of physical rehabilitation programmes or exercise programmes. Physical exercise plays a key role in speeding recovery compared with usual care. Undoubtedly, this recovery entails a greater ability to cope with the activities of daily living, probably contributing to physical and mental well‐being in older patients with pos‐COVID‐19 syndrome. However, areas for improvement, such as ventilatory efficiency and specific muscle group targeting, need further investigation to optimize intervention strategies.

This study has limitations that should be considered. An intention‐to‐treat analysis was applied. In this study, sample attrition (illness, dropout, etc.) was a concern during the rehabilitation programme and assessments of cardiorespiratory and muscular fitness, and body composition. Once older patients with post‐COVID‐19 sequelae were included in the study, it was not possible to know the causes of patient abandonment. Our interpretation of the results could be imprecise as we cannot exclude the possibility of completion or self‐selection bias. The missing cases were kept in the final analysis taking into consideration that they increase the variance of the estimated effects to a greater extent than the cases that are present for all measures. These factors could influence the final analysis and interpretation of the data. In addition, variability in participant characteristics can significantly influence results, particularly if there are notable differences in hospitalization and ventilation rates. Older patients in the group that underwent the telerehabilitation programme were hospitalized for longer and had greater ventilatory difficulties compared with the control group (see Table 2). However, the findings obtained from the post‐test evaluations highlight the key role of the telerehabilitation programme in significantly improving cardiorespiratory and muscular fitness and body composition, especially among older patients with prolonged hospitalizations and more severe ventilatory conditions.

Future research should focus on refining rehabilitation approaches and exploring novel interventions to address the diverse needs of post‐COVID‐19 patients and improve long‐term recovery outcomes. In this sense, older people and various populations with other types of diseases with similar consequences of fatigue, muscular and cardiorespiratory deconditioning (sarcopenia, chronic fatigue syndrome, chronic obstructive pulmonary disease, etc.) could be favoured by telerehabilitation programmes. Further research on participants' subjective satisfaction with this telerehabilitation programme will be essential for a deeper understanding of its effectiveness. Exploring their experiences and overall satisfaction will inform future improvements in telerehabilitation protocols and improve patient‐centered care in clinical practice. In addition, the advantages of the telerehabilitation programme could have been extolled if, for example, a group that did a traditional rehabilitation programme had been added. More research is necessary to clarify such assumptions.

## Conclusions

Several conclusions can be drawn from the implementation of a 12‐week rehabilitation programme in older people with post‐COVID‐19 syndrome. Cardiorespiratory fitness improved by increasing exercise capacity and power output during a cardiopulmonary exercise test. Muscular fitness improved by increasing leg extension muscular power output. In addition, body composition improved by increasing the fat free mass, soft lean mass, and skeletal muscle mass.

Contrary to our expectations, the telerehabilitation programme was not more effective in increasing *V̇*O_2peak_ and leg flexion power output and peak torque than the daily living activities of the control group.

## Funding

This study was funded by Consejo Superior de Deportes, Ministerio de Cultura y Deporte, convocatoria ‘Ayudas para proyectos de investigación en ciencia y tecnología aplicada a la actividad física beneficiosa para la salud (AFBS) y la medicina deportiva. EXP_74866’. The authors certify that they have complied with the ethical guidelines for authorship and publishing in the *Journal of Cachexia, Sarcopenia and Muscle*.^41^

## Conflict of interest

The authors declare that they have no competing interests.
